# Books and Pamphlets Reviewed

**Published:** 1863-10

**Authors:** 


### BOOKS AND PAMPHLETS REVIEWED.

*A Manual of Instruction for Enlisting and Discharging Soldiers, with special reference to the Medical Examination of Recruits, and the detection of disqualifying and feigned diseases. By* Robert Bartholow, A. M., M. D., *Assistant Surgeon U. S. Army, Surgeon in charge of McDougall General Hospital, Professor of Mil. Med. Jurisprudence, Army Medical School. Adopted by the Surgeon-General for issue to Medical Officers of the Army. Philadelphia:* J. B. Lippincott & Co., 1863. *For sale by Breed, Butler & Co.*

This book appears to us to be at the present time one of the most important of works. There is always importance to be attached to a well written book, but at the present time when physicians in many instances not thoroughly familiar with all the evidences of disease or acquainted with what really constitutes disability for military duty, have been appointed to make final decision upon the ability of men drafted into the military service, it is opportune and altogether indispensable that some work which is concise, correct and complete should be available. There has never been a duty imposed upon the medical officers of the army so difficult to perform with exact justice to both the government and the conscript, as the examination of drafted men. It requires for its proper performance a thorough medical education made complete by extensive practice, as well as experience in the examination of recruits; and we think that even then, errors in judgment would not be uncommon. It was never difficult to decide with tolerable exactness upon those who wanted to join the service as volunteers, for they had no diseases they desired to magnify, but men forced into the service are feeble, sickly, rheumatic, catarrhal, consumptive, insane, epileptic, deaf, blind, and in short, every way disabled. The author of this book has considered every topic, and given tests for feigned diseases, which are worthy the consideration of all examining boards. We are only sorry that the work had not been issued sooner, and read attentively long since; it would have been better, much better, for all concerned. As it is, and it is impossible that it should be otherwise, we will conclude our brief notice of a book which should be in the hands of every medical officer, by saying that it is well and carefully written, every important subject has received due attention, and the work shows great practical ability and careful observation in the author, who has had ample opportunity, and has given the profession a work upon the examination of recruits and detection of feigned diseases worthy the careful consideration of every medical officer of the army.

---

*What is the Modus Operandi of Medicines? Do they produce their effects by their Action on the Blood, as taught by all Modern Physiologists? or, do they produce their effects by their Action on the Organic Nervous System, through the agency of the Blood? The latter question considered and affirmatively answered. By* JOHN O'REILLY, M. D., F. R. C. S. I.

The position taken by the author of this pamphlet is, that all medicines exert their influence upon the system through the "organic nervous system." The ancients supposed that life was located in, or rather that the pineal gland was the seat of llfe. Dr. O'Reilly demonstrates that it is in the organic nervous system. We will make a short quotation, allowing the Doctor to speak, and our readers to judge for themselves. The pamphlet is certainly worthy a careful perusal:

"If it is true life can be destroyed by a blow on any of the principal "ganglions of the organic nervous system, as for instance the semilunar "ganglion, then it follows that life is located in the ganglions or organic "nervous system and not in the blood.

"If life is located in the organic ganglions, then it follows that life must "be located in the organic nervous system. If organic nerves are in the "coats of the arteries, then it follows that life must be located in the organic "nerves which supply the arteries. It has been stated by Prof. Dalton, "that too much importance is attached to the organic nervous system, as

" regards the operation of medicines, but if the statements I have made are " true, and I think their truth cannot be questioned, it follows that all the " operations of medicinal agents for good or evil, depend on their action on " the organic nervous system when introduced in the blood. When poisons " are introduced into the blood by the lacteal and lymphatics in the intes- " tinal canal, or by the lymphatics on the surface of the body, or by the " process of inhalation, the action is all the same, they destroy life by " destroying vitality in the organic nervous system.

" Blood-poisoning is a vague term, signifying that the blood has been " poisoned, but gives no explanation or idea how the poison causes death. " The blood carries the poison and communicates it to the organic glands " at the termination of the arteries.

" To illustrate the truth of this explanation, it is only necessary to call " the attention to what happens when arsenic is applied to a cancerous ulcer " on the lower extremity. The arsenic is absorbed by the lymphatics, it is " carried into the venous circulation to the right side of the heart, to the " lungs, to the left side of the heart, and by the aorta, its branches, ramifi- " cations, and capillaries, to all the organic glands wherever located. The " organic glands of the stomach become imbued with the poison; inflam- " mation of the mucous membrane of the stomach is the result, and symp- " toms of poisoning by arsenic present themselves,

" When Fowler's solution is given in a case of eczema, it is taken up by " the lacteals and lymphatics in the intestinal canal, passes into the venous " circulation, next into the arterial circulation, is given off to the organic " glands on the surface of the body, and changes the diseased action of the " organic glands, sometimes causing them to assume a healthy action.

" I will conclude by remarking, that the dilatation and increase in size of " the carotid arteries during the growth of the stag's horns, the dilatation " and increase in size of the uterine arteries during gestation, cannot be " attributed to the blood, or cannot be attributed to any substance intro- " duced into the blood, inasmuch as the other arteries continue in their " normal state. The dilatation and increase in size are attributable to what " is called vital action, but as vital action is synonymous with life, and as " life is located in the organic nervous system, it follows, therefore, that " dilatation and increase in size of the carotids and uterine arteries are due " to the presence of the organic nerves in the coats of the arteries."

*On Artificial Dilatation of the Os and Cervix Uteri by Fluid Pressure from above; a reply to Drs. Keiller of Edinburgh, and Arnott and Barnes of London.* *By* HORATIO R. STORER, M. D., *of Boston, Surgeon to the Pleasant St. Hospital for Women, Member of the Obstetric and Medico-Chirurgical Societies of Edinburgh, etc. Re-printed from "The Boston Medical and Surgical Journal," for July* 2, 1862.

This is an article considering the subject of "Dilatation of the Os and Cervix Uteri" in obstetric surgery. It is mainly devoted to the evidences of priority of discovery, and practical application of "fluid pressure from above," in accomplishing this result. He says:

"Immediately on entering practice, it became evident to me that the great field for advance in obstetric therapeutics was *the interior* of the uterus—an opinion that was daily strengthened during the intimate relations to which I was admitted by Prof. Simpson in 1854–55.

"At that time the sole means, at all safe and reliable, of directly reaching the interior of the unimpregnated uterus, was by the use of expansible tents, then only made of sponge, first suggested for this purpose by Simpson in 1844. It is true, that for the induction of premature labor, for which the method to be considered was first proposed to the profession by both Dr. Keiller and myself, and for which its use is now urged by such competent authority abroad, there had been many measures suggested and practiced—all of them, however, acting either secondarily or by reflex action, as do galvanism, mammary irritation, puncture of the membranes, their separation from the uterus by bougies, the injection of water or air, an agent here so dangerous, or by the uterine sound, and also, there seems to me good reason for believing, the so-called oxytoxics, as ergot of rye; or by a stimulating or dilating force applied and first acting from below.

"These remarks apply with equal force to all methods that had then been proposed—to those of Hamilton and Hopkins; to the flexible catheter left in the cavity of the uterus by Merrem, Krause and Simpson; to the vaginal and cervical plugs and dilators of Brünninghausen, Osiander, Von Busch, Hüter, Gariel and Braun; to the carbonic acid douche, suggested Brown-Séquard, and so fatal in the hands of Scanzoni and others; and to the water douches, of Kiwisch, applied to the vagina, and of Schweighaüser and Cohen, to the uterine cavity. These several means, while they were applicable but partially and with varying success to the pregnant uterus, were wholly unfitted, with the exception of sponge tents, for opening up that which already contained no fœtus; and for this the elastic bougies of McIntosh and the unyielding ones of Simpson, the spring-knife of the latter, the hollow tubes used by Wakely for urethral stricture and adopted from him by Baker Brown, and the instruments of Rigby, Graham, Weir, Osiander, Busch, Krause and Jobert, with expanding metalic blades, are either insufficient or attended with too much hazard.

"Caoutchouc bags or sacs, distended with air, had been proposed some years previously by Gariel, for the treatment of displacements of the uterus by pressure from below, and for plugging the vagina in cases of hæmorrhage. He had also suggested their possible introduction into the cervix, not, however, through it, for the purpose of overcoming stricture of that canal, and had even asked, "if this property of the bulbous air sound could not be turned to advantageous use in inducing premature labor?" here referring however, to their use in the vagina, as had already been suggested by Hüter and Braun, were attended with singularly unfortunate results, Breit and others reporting a mortality of six patients out of fourteen.

"To sponge tents applied to the cervix there attach, as I have already intimated, various important objections. They are readily acted upon chemically by the uterine and vaginal secretions, and from their organic character quickly undergo putrefactive decomposition, subjecting the patient to a certain amount of risk from such possible absorption as is hereby implied. They act at times with great rapidity and force, and where the tissues are morbidly friable, if not very carefully made, they may produce unintentional or dangerous tension and laceration.

"From direct experience of these several dangers, it became my aim to find, if possible, a substitute for sponge in the dilatation of the cervix, and in May, 1855, in a paper read before the Medico-Chirurgical Society of Edinburgh, I proposed the use of tents prepared from the bark of our indigenous slippery elm. Shortly after, during the publication of Dr. Simpson's Memoirs, I had again occasion to refer to the disadvantage of sponge under certain circumstances, and at still greater length in a paper presented during the fall of the same year.

"The use of elm tents in my own hands and those of others who have communicated with me upon the subject, proved the agent greatly superior to sponge in those cases where a low and moderate action is desired, as, for instance, in mechanical dysmenorrhœa and certain forms of sterility; and as yet I know of nothing that will here better answer the indication although during the course of my experiments in this direction I have tried a variety of other substances, as althea root, etc., among them the root of gentian, afterwards made the basis of a memoir upon mucilaginous tents, by an English Surgeon, Dr. Aveling, of Sheffield, in apparent ignorance of his having been anticipated by my suggestion of three years before.

"Steadily pursuing these efforts towards the solution of the interesting problem proposed, I again called the attention of the profession to its importance by a paper published in Philadelphia early in 1859, in which were pointed out the several indications for artificial dilatation of the cervix uteri, and the several dangers attaching thereto, alike in the induction of premature labor, the assistance of the progress of accidental abortion and of labor at the full time, the exposure of the uterine cavity for the purposes of diagnosis and treatment, both in diseases puerperal and non-puerperal.

"During the preparation of this paper, duly appreciating, as will be apparent from its perusal, the actual and relative value in the assistance or induction of labor, of the several elements of action involved—namely, dilatation of the cervical canal, detachment of the membranes from the walls of the uterus, and the prolonged preservation intact of the bag of waters—I had frequent conversations upon the subject with my friend Dr. Nathan Hayward, of Roxbury, now Surgeon of the 20th Massachusetts Regiment, and at that time associated with me in the conduct of the Eustis St. Dispensary. With his assistance, I contrived an instrument designed to combine the various indications just referred to, and this was used in practice upon the first favorable case that presented itself to us, on April 13, 1859. The operation was entirely successful; labor was prematurely induced at the eighth month in a woman who had four times previously undergone craniotomy, and a living male child was delivered. The case was the more interesting to us from the fact that both Dr. Hayward and myself were present at her last confinement; I had turned and delivered the trunk, but it was found absolutely necessary to lessen the head from below before it could be made to pass.

"The proposal of the measure now resorted to, as I supposed, for the first time, was made at considerable length under the name of 'the uterine dilator,' and the case reported in July, 1859. I then stated that the instrument, introduced within the cavity of the uterus, produced its action in a three-fold manner: "reflexively, as a foreign body; reflexively and directly, by separating the membranes from the uterine walls; and directly, as a fluid wedge, by dilating the os; in each of these three respects, its effect being in proportion to the amount of distension applied. It should be noticed that this dilatation," I also added, "is *from above downwards*, while the tent dilates from below upwards." I referred to the similarity of this instrument to one suggested for the female urethra by Spencer Wells, of London, some months previously, which in its turn had been taken from a modification by Thompson of James Arnott's urethral dilator, so forcibly brought forward as long back as 1818, both by myself and his brother Neil, and shortly after by Ducamp in a memoir that received much approbation from the French Academy. I mentioned, also, the curious fact in the history of the various means that have been proposed for dilatation of the uterus, that they have all, without exception, been based, directly or indirectly, upon some method previously in use for the treatment of strictures of the male urethra.

"I have thus plainly stated my own position in relation to the plan of dilating the uterus by fluid pressure acting from above, and have shown the gradual and successive steps by which I arrived at the idea and its development. The medium employed for dilating my sac was water; to the dangers of air used for this purpose, as it has been by others who have taken part in this controversy, I then called attention, as I shall again do in the course of the present communication.

"Now as to opposing claims, I shall endeavor to state them as fairly, even at my own expense."

